# Reply to comments on “Anatomical and visual outcomes of fovea-sparing internal limiting membrane peeling with or without inverted flap technique for myopic foveoschisis”

**DOI:** 10.1186/s12886-023-02986-2

**Published:** 2023-06-14

**Authors:** Dezhi Zheng, Zijing Huang, Weiqi Chen

**Affiliations:** Joint Shantou International Eye Center of Shantou University, Chinese University of Hong Kong, 69# North Dongxia Road, Jinping District, 515041 Shantou, Guangdong P.R. China

We express our gratitude to the concerns raised by Hsieh et al. regarding to our investigation on the anatomical and visual outcomes of fovea-sparing internal limiting membrane (ILM) peeling with or without the inverted flap technique for myopic foveoschisis (MF).

In our study, we incline to interpret the case mentioned by Hsieh et al. as an occurrence of postoperative full-thickness macular hole (FTMH) formation. FTMH is characterized by an interruption of all neural retinal layers from the ILM to the retinal pigment epithelium (RPE) [1]. We concur that the inverted ILM flap has the benefit of averting the FTMH formation by preventing the direct damage to the foveal structure. The postoperative diameter of foveal neural retinal layers interruption in the case mentioned by Hsieh et al. enlarged from 151 μm at one week after surgery to 597 μm at one month later despite the presence of the inverted ILM flap still covering the fovea. Remarkably, the postoperative FTMH in our study, in which the inverted ILM flap was utilized, spontaneously closed without further intervention. This suggests that the inverted flap could provide a better protective effect on the macula, even in cases where postoperative FTMH formation occurs.

Previous studies have reported the incidence of FTMH development subsequent to fovea-sparing ILM peeling in myopic traction maculopathy ranging from 5.6 to 9.7%. The presence of preoperative lamellar macular hole (LMH) is also associated with an increased risk of postoperative FTMH formation [2, 3]. We agree with Hsieh et al. that the increased parafoveal traction, resulted from scleral staphyloma and inner retina relative deficiency, may contribute to the postoperative FTMH formation. In our study, the two eyes, which experienced the postoperative FTMH formation, were both accompanied by severe preoperative LMH, and the FTMH occurred with the restoration of MF.

The inverted ILM flap technique has been commonly used for FTMH. A flap of ILM covering the surface of macular hole promotes the proliferation of glial cells and contributed to the restoration of foveal architecture as a scaffold [4]. The flap closure configuration, characterized by only a thin layer of the inverted ILM closed the FTMH, is observed in 14 to 16% of eyes received the inverted ILM flap technique [5, 6]. Such flap closure is more commonly observed in large macular holes and is associated with lower postoperative visual acuity as compared to the eyes with U-type or V-type closure [5, 7]. Fundamentally, flap closure refers to a situation where the FTMH remains open with inverted ILM covering. However, the incidence of postoperative FTMH formation may be underestimated in case without preoperative FTMH if the flap closure configuration is overemphasized.

The satisfactory success rate of the operation has been shown in our study. That only one eye in each group of the inverted and non-inverted flaps experienced a delayed onset of postoperative FTMH. Our findings were based on the statistical analyses from the retrospective clinical case observation. Further prospective controlled trials with larger sample size are warranted to evaluate the advantages of the inverted ILM flap technique for MF comprehensively.

## Data Availability

Not applicable.
